# Cardiovascular disease patients have increased risk for comorbidity: A cross-sectional study in the Netherlands

**DOI:** 10.1080/13814788.2017.1398318

**Published:** 2017-11-23

**Authors:** Candan Kendir, Marjan van den Akker, Rein Vos, Job Metsemakers

**Affiliations:** ^a^ Department of Family Medicine, Dokuz Eylul University Faculty of Medicine Izmir Turkey; ^b^ Department of Family Medicine, School CAPHRI, Maastricht University Maastricht the Netherlands; ^c^ Academic Center for General Practice/Department of Public Health and Primary Care, KU Leuven Leuven Belgium; ^d^ Department of Methodology & Statistics, School CAPHRI, Maastricht University Maastricht the Netherlands; ^e^ Department of Medical Informatics, Erasmus Medical Centre Rotterdam the Netherlands

**Keywords:** Comorbidity, cardiovascular diseases, chronic diseases

## Abstract

**Background:** Comorbidity is a cause of increased mortality, decreased quality of life and increased use of healthcare services. It is important particularly for physicians and other healthcare providers in primary care settings to evaluate these patients properly. Cardiovascular diseases (CVD) are the most common cause of death from non-communicable diseases worldwide and are characterized by a high level of comorbidities.

**Objectives**: To address the distribution of CVDs and comorbidities across sociodemographic groups and associations between CVDs and comorbidities.

**Methods:** A cross-sectional study was conducted using data of 67 786 patients. Data were collected by the Registration Network Family Practices (RegistratieNet Huisartspraktijken, RNH). Comorbidities were analysed using chi-square and logistic regression analyses.

**Results:** At the time of study, 26.5% of the patients had at least one CVD and 10.5% of patients had two or more CVD diagnoses. The strongest association within cardiovascular diseases were between health failure and arrhythmias (OR: 9.20; 95%CI: 7.78–10.89). Coronary artery disease and hypertension had strong relationship with diabetes (OR: 2.22; 95%CI: 2.02–2.45, OR: 2.22; 95%CI: 2.02–2.45 respectively) and lipid metabolism disorders (OR: 2.04; 95%CI: 1.87–2.23, OR: 2.04; 95%CI: 1.87–2.23, respectively). The strongest associations for cerebrovascular diseases were with epilepsy (OR: 4.09; 95%CI: 3.29–5.10) and arrhythmias (OR: 2.23; 95%CI: 1.99–2.50).

**Conclusion:** One out of every four patients suffered from at least one CVD. Having one CVD increased the risk of another, co-occurring CVD and a higher number of other chronic diseases.

KEY MESSAGES

- 26.5% of patients had ≥1 CVD; 10.5% had ≥2 CVD diagnoses.
- Each CVD was associated with other CVDs and many other comorbidities.
- In case of CVD, physicians should be alert for another CVD; these patients must be closely and carefully monitored for other chronic diseases.

## Introduction

Cardiovascular diseases (CVDs) are the most common cause of death from non-communicable diseases. Globally, an estimated 17.5 million people died from CVDs in 2012, representing 31% of all global deaths [1]. According to the ‘Top 10 leading causes of death by age groups’ statistics of the Center for Disease Control (CDC), CVDs are in the top three of most common causes of death in people older than 35 years [2]. Weiner et al., found that patients with CVD who also have chronic kidney disease are 35% more vulnerable to recurrent CVD or mortality compared to CVD patients without comorbidity [3]. Tripathy et al., found that the risk of mortality from CVD among patients who have no history of previous heart attack is higher for patients with diabetes than for those without diabetes [4].

It is important for GPs and other healthcare providers to manage patients with CVD in a way that takes comorbidity into account [5]. Management of these patients in primary care settings is important since patients with comorbidity are characterized by increased mortality, decreased quality of life, and increased use of healthcare services compared to patients without comorbidity [6–8]. In addition, the occurrence of comorbidity influences medical decision-making by physicians as regards prevention, treatment and utilization of services [9], and comorbidity increases the frequency of visits to general practitioners (GPs) and other medical specialists [10].

Previous studies have established relationships between CVDs and other chronic conditions, mainly focusing on specific age groups or chronic disease types [11–14], but few studies have addressed an extensive range of chronic disease categories [8,15,16]. The healthcare providers in general practice should be alert for additional diseases in patients with CVD, and should monitor these patients closely and spend more time with them. This can help to prevent comorbidity or slow down disease progression over time, and might result in higher quality of life and decreased mortality [10]. It is against this background that the present study intended to help primary care providers to optimize the care for patients with CVD by describing the relationships between CVDs and comorbidity.

This study aimed to explore the occurrence of comorbidity among patients with CVD.

## Materials and methods

### Study design and participants

This cross-sectional, observational study was based on data from the Registration Network Family Practices (RegistratieNet Huisartspraktijken, RNH). This network was developed in the Netherlands in 1988 to establish a computerized database. Basic sociodemographic characteristics of patients and all relevant past and current permanent health problems are recorded systematically and are updated continuously. Registration of medical information is part of daily routine. Registered health problems are added and uploaded to the RNH database every three months. For the registration of diseases, the International Classification of Primary Care (ICPC) is used, applying the criteria of the International Classification of Health Problems in Primary Care (ICHPPC-2), or other relevant criteria.

Once a GP accepts inclusion onto the RNH system, patients are informed about their GP’s participation in the RNH-system and can opt-out. Sixty-five GPs from 22 practices store information and all of them are located in the province of Limburg in the Netherlands. The patient population in the database is representative for the Dutch population [17].

As of 31 December 2015, 69 953 patients were registered in the database. Selecting for age (<85 years) and excluding patients with data errors, 67 786 patients were included in the analysis.

### Sociodemographic and lifestyle variables

The RNH database records permanent, serious, chronic or recurrent problems, together with a limited number of sociodemographics like age, sex, and living situation. For our analysis, the age of the patients was stratified into four age groups; young (0–24), adult (25–44), middle age (45–64) and older (65–84). In addition, the patients’ living situation was classified into five categories: living with a family, single, as a couple without children, living in nursing home or other, such as commune, and unknown.

*Disease groups.*The most common CVDs were selected and clustered into five groups based on pathophysiological similarities, before relating them to the chronic non-cardiovascular diseases. The subgroups of both CVD groups and non-cardiovascular chronic disease groups are shown in Supplementary Appendix 1 (available online). Only diseases affecting the heart or arterial blood vessels were included in the CVD groups, whereas diseases affecting the venous system were excluded as index diseases but included as comorbidities. Thus, arrhythmias were included as a chronic comorbidity disease group. In total, nine CVDs are grouped into five disease groups. These are: (1) acute myocardial infarction and other/chronic ischaemic heart disease such as coronary artery disease; (2) heart failure; (3) uncomplicated hypertension and hypertension with involvement in target organs; (4) cerebrovascular disease consisting of transient ischaemic attack and stroke/cerebrovascular accident; and (5) peripheral artery disease atherosclerosis and other arterial diseases.

The chronic disease groups were based on the list previously used by Aarts et al. [12]. However, we made some modifications to study the relationship between selected CVDs and additional chronic diseases. A few diseases (osteoarthritis, rheumatoid arthritis, osteoporosis, epilepsy, lipid metabolism disorders, asthma, and COPD/chronic bronchitis) were analysed separately to examine the relationship of each disease with CVDs. A few other disease categories (other diseases of the nervous system, other chronic respiratory diseases, chronic skin ulcer) were deleted because of the low number of patients in these groups and lack of statistical power.

In the study, CVD was used as the index disease and an additional disease to CVD is accepted as comorbidity.

### Statistical analysis

The statistical analysis was done using SPSS 22.0 software. The relationship between sociodemographics and having none, one, or two or more CVDs, and the difference between observed and expected probabilities, were studied using chi-square analysis. A *P*-value of less than 5% was considered statistically significant. The relationship between each CVD and another CVD or with another chronic comorbid disease was analysed by logistic regression, involving both crude analysis and adjustment for age and sex. In these analyses, age was used as a categorical variable. We also analysed the relationship between having two or more CVDs and the numbers of comorbidities (none, one or two, and more) among patients with CVD, using logistic regression adjusted for age and sex. The results are presented as odds ratios with 95% confidence intervals or Pearson chi-square values.

## Results

### Characteristics of the study population

The mean age of the patients was 45 ± 21.7 years with a median age of 48 years; descriptive data of patients are shown in Table 1. At the time of study, 26.5% of the patients had at least one CVD and 10.5% of patients had two or more CVD diagnoses. The proportion of patients who had CVD as well as the number of CVDs per patient increased with increasing age.

**Table 1.** Description of the study population (*n* = 67786).

	Cardiovascular diseases			
	None % (*n*)	1 % (*n*)	≥2 % (*n*)	Total % (*n*)
Age groups				
0–24	95.6 (14365)	4.0 (595)	0.4 (66)	100 (15026)
25–44	92.7 (14327)	6.2 (960)	1.1 (166)	100 (15453)
45–64	70.0 (15700)	20.9 (4699)	9.1 (2044)	100 (22443)
65–84	36.5 (5430)	30.7 (4567)	32.7 (4867)	100 (14864)
Sex				
Male	72.8 (24293)	15.2 (5059)	12.0 (4011)	100 (33363)
Female	74.2 (25529)	16.7 (5762)	9.1 (3132)	100 (34423)
Living situation				
Family	74.3 (26899)	16.1 (5821)	9.6 (3474)	100 (36194)
Single	68.2 (5009)	18.0 (1318)	13.8 (1014)	100 (7341)
Couple	58.9 (4921)	22.5 (1877)	18.7 (1561)	100 (8359)
Other	77.2 (358)	12.3 (57)	10.6 (49)	100 (464)
Unknown	81.9 (12635)	11.3 (1748)	6.8 (1045)	100 (15428)
Total	73.5 (49822)	16.0 (10821)	10.5 (7143)	100 (67786)

Supplementary Appendix 2 shows the distribution of other chronic diseases over the different age groups, sex and living situation. In nearly all chronic disease groups, the number of cases increased greatly among persons aged over 45 years. The most common diagnoses were diseases of the eye, diseases of the veins, osteoarthritis, mood disorders, asthma and lipid metabolism disorders. One third of the patients had more than one non-CVD diagnosis.

### Comorbidity with CVDs

Table 2 shows the associations between CVDs and other CVDs. For all CVD groups, positive and significant odds ratios were found for all combinations, which means that having one CVD always increased the risk of having another CVD.

**Table 2.** Relationship of cardiovascular diseases with other cardiovascular diseases.

	Coronary artery diseases	Hypertension	Heart failure	Cerebrovascular diseases	Peripheral artery diseases
	Adj. OR (95%CI)	Adj. OR (95%CI)	Adj. OR (95%CI)	Adj. OR (95%CI)	Adj. OR (95%CI)
Diseases					
Peripheral artery disease	3.11 (2.74–3.53)	2.25 (2.03–2.49)	3.64 (2.96–4.47)	3.59 (3.16–4.09)	–
Cerebrovascular diseases	1.73 (1.52–1.96)	2.26 (2.07–2.48)	2.25 (1.81–2.78)	–	^a^
Heart failure	6.13 (5.14–7.31)	2.06 (1.75–2.43)	–	^a^	^a^
Hypertension	1.90 (1.75–2.07)	–	^a^	^a^	^a^
Coronary artery disease	–	^a^	^a^	^a^	^a^
Additional cardiovascular diseases	2.30 (2.11–2.51)	2.11 (1.98–2.24)	3.44 (2.78–4.25)	2.58 (2.33–2.84)	3.29 (2.94–3.69)

^a^ Changing dependent and independent variables resulted in same odds ratios.

Adj. OR: odds ratios adjusted for age and sex; 95%CI: 95% confidence interval.

Table 3 shows the associations between CVDs and other chronic diseases. All CVDs showed associations with many of the comorbidities studied.

**Table 3.** Relationship between cardiovascular diseases and chronic comorbid diseases.

	Coronary artery diseases	Hypertension	Heart failure	Cerebrovascular diseases	Peripheral artery diseases
Chronic comorbid diseases	Adj. OR (95% CI)	Adj. OR (95% CI)	Adj. OR (95% CI)	Adj. OR (95% CI)	Adj. OR (95% CI)
Diseases					
All malignancies	1.30 (1.15–1.47)	1.19 (1.10–1.29)	1.55 (1.25–1.92)	1.27 (1.12–1.45)	1.27 (1.10–1.47)
Peptic ulcers	1.41 (1.18–1.68)	1.26 (1.11–1.43)	1.95 (1.46–2.62)	1.60 (1.33–1.94)	2.05 (1.69–2.48)
Diseases of the eye	1.51 (1.37–1.67)	1.68 (1.58–1.79)	2.34 (1.98–2.78)	1.61 (1.45–1.79)	1.82 (1.62–2.04)
Diseases of the ear	1.41 (1.25–1.59)	1.36 (1.25–1.48)	1.61 (1.30–2.00)	1.54 (1.35–1.75)	1.20 (1.03–1.41)
Arrhythmias	2.55 (2.30–2.82)	2.05 (1.90–2.21)	9.20 (7.78–10.89)	2.23 (1.99–2.50)	1.96 (1.72–2.23)
Pulmonary circulatory disease	1.33 (1.09–1.62)	1.30 (1.14–1.48)	2.10 (1.54–2.86)	1.80 (1.48–2.18)	1.74 (1.40–2.16)
Osteoarthritis	1.34 (1.22–1.47)	1.65 (1.56–1.75)	1.45 (1.22–1.72)	1.29 (1.17–1.43)	1.48 (1.32–1.65)
Rheumatoid arthritis	1.33 (1.07–1.65)	1.41 (1.23–1.61)	2.15 (1.54–2.99)	1.65 (1.33–2.03)	1.38 (1.07–1.77)
Mood disorders	1.46 (1.30–1.62)	1.32 (1.23–1.40)	1.53 (1.24–1.89)	1.58 (1.41–1.77)	1.38 (1.21–1.57)
Other mental disorders	1.49 (1.28–1.74)	1.14 (1.04–1.25)	1.68 (1.26–2.25)	1.57 (1.33–1.84)	1.52 (1.28–1.82)
COPD and bronchitis	1.97 (1.74–2.22)	1.34 (1.23–1.46)	3.27 (2.69–3.97)	1.78 (1.56–2.04)	3.06 (2.68–3.48)
Psoriasis	1.42 (1.21–1.67)	1.32 (1.19–1.46)	1.61 (1.19–2.16)	1.34 (1.12–1.60)	1.58 (1.31–1.90)
Thyroid diseases	1.81 (1.54–2.13)	1.42 (1.29–1.57)	2.14 (1.64–2.78)	1.45 (1.23–1.72)	1.46 (1.21–1.76)
Diabetes mellitus	2.22 (2.02–2.45)	3.17 (2.96–3.40)	3.16 (2.66–3.75)	1.68 (1.50–1.87)	2.24 (1.99–2.52)
Lipid metabolism disorder	2.04 (1.87–2.23)	3.53 (3.34–3.74)	1.29 (1.08–1.54)	1.58 (1.43–1.74)	2.06 (1.86–2.29)
Gout	1.53 (1.32–1.77)	3.22 (2.89–3.60)	3.11 (2.46–3.93)	1.61 (1.36–1.91)	1.88 (1.58–2.25)

Adj. OR: odds ratios adjusted for age and sex; 95%CI: 95% confidence interval.

Coronary artery diseases had the strongest association with arrhythmias (OR: 2.55; 95%CI: 2.30–2.82), diabetes mellitus (OR: 2.22; 95%CI: 2.02–2.45) and lipid metabolism disorder (OR: 2.04; 95%CI: 1.87–2.23).

Hypertension showed a strong association with arrhythmias (OR: 2.05; 95%CI: 2.30–2.82), diabetes mellitus (OR: 2.22; 95%CI: 2.02–2.45), and lipid metabolism disorder (OR: 2.04; 95%CI: 1.87–2.23).

Heart failure showed the strongest association with arrhythmias (OR: 9.20; 95%CI: 7.78–10.89). In addition, it showed a strong association with diseases of the eye (OR: 2.34; 95%CI: 1.98–2.78), pulmonary circulatory disease (OR: 2.10; 95%CI: 1.54–2.86), rheumatoid arthritis (OR: 2.15; 95%CI: 1.54–2.99), COPD and bronchitis (OR: 3.27; 95%CI: 2.69–3.97), thyroid disorders (OR: 2.14; 95%CI: 1.64–2.78), diabetes mellitus (OR: 3.16; 95%CI: 2.66–3.75) and gout (OR: 3.11; 95%CI: 2.46–3.93). Alternatively, heart failure showed a negative association with migraine and headache (OR: 0.57; 95%CI: 0.35–0.93).

Cerebrovascular diseases showed a positive association with all chronic comorbidities, the strongest associations being those with epilepsy (OR: 4.09; 95%CI: 3.29–5.10) and arrhythmias (OR: 2.23; 95%CI: 1.99–2.50).

Peripheral artery diseases showed a strong association with peptic ulcer (OR: 2.05; 95%CI: 1.69–2.48), COPD and bronchitis (OR: 3.06; 95%CI: 2.68–3.48), diabetes mellitus (OR: 2.24; 95%CI: 1.99–2.52) and lipid metabolism disorder (OR: 2.06; 95%CI: 1.86–2.29).

## Discussion

### Main findings

Our findings show that in a large population of patients from a primary care registration network (RNH), one out of every four patients suffered from at least one CVD. Having one CVD increased the risk of another, co-occurring CVD and a higher number of other chronic diseases. The strongest association was found between heart failure and coronary artery disease. In addition, patients with peripheral artery disease were found to have a more than three-fold increased risk for coronary artery disease, heart failure and cerebrovascular disease compared to patients without peripheral artery disease. Diseases of the eyes, osteoarthritis, mood disorders and lipid metabolism disorders were the most prevalent comorbidities, and all showed a positive association with each of the CVDs.

### Comparison with existing literature

This study is a modified replication of the 1997 study by Landwehr Johan et al. [15], who analysed the co-occurrence of six CVD groups with 30 chronic disease groups in the RNH population (*n =* 73 254). We analysed five CVD groups and 25 chronic disease groups, using the RNH database as available 19 years later. Comparing the results of these two studies shows that the current study found a higher number of comorbidities with a significant positive association with CVDs. Furthermore, the associations between CVDs and comorbidity we found were stronger than those in the previous study when making the same adjustments. This might be a result of increased and improved screening or diagnostics of some diseases like diabetes mellitus, lipid metabolism disorders or epilepsy, resulting in earlier treatment with better results and less comorbidity. The difference could also be due to some disease groups being analysed separately in the previous study and grouped together in our study. However, having more comorbidity compared to the previous study is also a result of aging population in Europe that has been shown on the dataset of Eurostat in comparison of years 2005 and 2015 [18].

Previous studies also found that patients with CVD were at increased risk for comorbidities compared to patients without such disease [4,19–21]. In our study, all CVDs showed a positive association with all malignancies, peptic ulcer, eye diseases, ear diseases, arrhythmias, pulmonary circulatory diseases, osteoarthritis, rheumatoid arthritis, mood disorders, other mental disorders, COPD and bronchitis, thyroid disorders, diabetes mellitus, lipid metabolism disorders and gout.

We found a negative association between migraine and heart failure, which was not described previously, to our knowledge. Based on the existing literature [15], we expected a positive association between migraine and all CVDs, but in our study, the association between migraine and CVDs was not significant for any of the CVD groups. We found a significant positive association only between migraine and hypertension and cerebrovascular diseases.

Another result of the present study which differed from previous work regarded the association between asthma and CVDs. Asthma showed a statistically significant positive association only with hypertension, heart failure and cerebrovascular diseases, not with other CVDs. In the literature, however, CVDs are the second most common comorbidities of asthma [22], so we had expected a positive association between asthma and each of the CVDs. This might be due to lack of questioning of the predisposing and associated factors such as smoking habits, family history and environmental factors.

### Strengths

- The RNH database includes a large number of patients and a broad spectrum of chronic disease diagnoses. Its dataset covers a long period, and the data are reliable.
- The study also included children, young adult patients, providing data on the whole population with CVDs and comorbidities, rather than being limited to the older population, who are known to have a higher number of comorbidities.

### Limitations

- Lack of availability of some sociodemographic data and risk factors, like body mass index (BMI) and patients’ smoking behaviour, limited us in adjusting our findings for potential confounders.
- We did not analyse the data for concordant (sharing the same pathogenesis or treatment approach) or discordant (not sharing the same pathogenesis or treatment approach) morbidity. Our study thus provides no indication whether comorbidities were a result of common pathological mechanisms.

## Conclusion

GPs and other primary care healthcare providers should take the results of the present study into account in making decisions regarding the care for patients with CVDs and comorbidities. Our findings have implications for the way healthcare providers manage and coordinate the care for these patients. Once a patient has a CVD, the healthcare provider should be particularly alert for another CVD, and must closely and carefully monitor these patients for other chronic diseases. In addition, knowledge of the association between CVDs and comorbidities can guide health promotion workers in developing guidelines for the management of these patients in primary care. Our findings also have implications for health legislators who have to define priorities for future healthcare planning. We expect that prevention, screening, early diagnosis and treatment of comorbid conditions in CVD patients will improve the health outcomes and quality of life for these patients.

## Supplementary Material

Supplementary Appendices 1-3
