# Supplementary material for: Cardiovascular disease patients have increased risk for comorbidity: A cross-sectional study in the Netherlands
Source: Eur J Gen Pract. 2017 Nov 23;24(1):45–50. doi: 10.1080/13814788.2017.1398318 (PMC5795764; doi:10.1080/13814788.2017.1398318)
Supplement: Supplementary Appendices 1-3 [file IGEN_A_1398318_SM0305.pdf]

Appendix 1: Disease groups

Disease groups	Diseases	Codes
All malignancies	Carcinomatosis	A79
	Hodgkin's disease	B72
	Leukaemia	B73
	Malignant neoplasm stomach	D74
	Malignant neoplasm colon/rectum	D75
	Malignant neoplasm pancreas	D76
	Malignant neoplasm other/unspecified	D77
	Neoplasm of eye/adnexa	F74
	Malignant neoplasm	N74
	Malignant neoplasm bronchus/lung	R84
	Other malignant neoplasm	R85
	Malignant neoplasm thyroid	T71
	Malignant neoplasm of kidney	U75
	Malignant neoplasm of bladder	U76
	Other malignant neoplasm urinary tract	U77
	Malignant neoplasm	W72
	Malignant neoplasm cervix	X75
	Malignant neoplasm breast	X76
	Other malignant neoplasms	X77
	Malignant neoplasm prostate	Y77
	Other malignant neoplasm	Y78
Peptic ulcers	Duodenal ulcer	D85
	Other peptic ulcers	D86
Other chronic gastrointestinal diseases	Diverticular disease	D92
	Chronic enteritis/ulcerative colitis	D94
	Cirrhosis/other liver disease	D97
Diseases of eye	Retinopathy	F83
	Macular degeneration	F84
	Blindness all degrees/types	F94
	Cataract	F92
	Glaucoma	F93
Diseases of ear	Otosclerosis	H83
	Deafness all degrees NOS	H86
	Presbycusis	H84
Arrhythmias	Atrial fibrillation/flutter	K78
	Paroxysmal tachycardia	K79
	Ectopic beats all types	K80
	Heart valve disease non-rheumatic NOS	K83
Pulmonary circulatory diseases	Pulmonary embolism	K93
	Phlebitis/thrombophlebitis	K94
Diseases of veins	Varicose veins of legs	K95
	Haemorrhoids	K96
Osteoarthritis	Osteoarthritis of spine	L84
	Osteoarthritis of hip	L89
	Osteoarthritis of knee	L90
	Other osteoarthritis	L91
Osteoporosis	Osteoporosis	L95
Acquired movement disorders	Acquired deformities of spine	L85
	Acquired deformities of limbs	L98
Rheumatoid arthritis	Rheumatoid arthritis/allied conditions	L88
Migraine and headache	Migraine	N89
	Cluster headache	N90
Epilepsy	Epilepsy all types	N88
Mood disorders	Depressive disorder	P76
	Affective psychosis	P73
	Anxiety disorder/anxiety state	P74
Other mental disorders	Other organic psychosis	P71
	Schizophrenia all types	P72
	Hysterical/hypochondriacal disease	P75
	Suicide attempt	P77
	Other neurotic disease	P79
	Personality disorder	P80
	Other/unspecified psychosis	P98
COPD and bronchitis	Chronic bronchitis/bronchiectasis	R91
	Emphysema/COPD	R95
Asthma	Asthma	R96
Atopic dermatitis	Atopic dermatitis/eczema	S87
Psoriasis	Psoriasis	S91
Thyroid disorders	Hyperthyroidism/thyrotoxicosis	T85

(*continued*)

Appendix 1. Continued

Disease groups	Diseases	Codes
	Hypothyroidism/myxoedema	T86
Diabetes mellitus	Diabetes mellitus	T90
Lipid metabolism disorder	Lipid metabolism disorder	T93
Gout	Gout	T92
Urinary calculus	Urinary calculus	U95
Cardiovascular disease groups		
Coronary artery diseases	Acute myocardial infarction	K75
	Other/chronic ischaemic heart diseases	K76
Heart failure	Heart failure	K77
Hypertension	Uncomplicated hypertension	K86
	Hypertension with involvement of target organs	K87
Cerebrovascular diseases	Transient cerebral ischaemia	K89
	Stroke/cerebrovascular accident	K90
Peripheral arterial diseases	Atherosclerosis excluding heart/brain	K91
	Other arterial obstruction/peripheral vascular disease	K92

Appendix 2: Prevalence of chronic comorbidities, stratified for age group, sex and living status

	Age groups				Gender	
Chronic comorbid diseases	0–24 % (*n*)	25–44 % (*n*)	45–64 % (*n*)	65–84 % (*n*)	Male % (*n*)	Female % (*n*)
All malignancies	0.6 (20)	5.2 (177)	31.4 (1064)	62.8 (2129)	41.3 (1401)	58.7 (1989)
Peptic ulcers	0.3 (3)	4.8 (57)	30.8 (364)	64.1 (757)	63.0 (744)	37.0 (437)
Other chronic gastrointestinal diseases	1.5 (49)	8.6 (277)	37.1 (1199)	52.9 (1711)	48.5 (1569)	51.5 (1667)
Diseases of the eye	0.6 (34)	2.4 (130)	20.1 (1081)	76.8 (4130)	46.3 (2490)	53.7 (2885)
Diseases of the ear	3.1 (93)	6.6 (197)	25.3 (751)	65.0 (1932)	56.1 (1667)	43.9 (1306)
Arrhythmias	3.1 (107)	6.1 (210)	27.4 (951)	63.5 (2202)	51.8 (1798)	48.2 (1672)
Pulmonary circulatory disease	1.6 (20)	9.1 (113)	39.3 (488)	50.0 (620)	44.7 (555)	55.3 (686)
Diseases of the veins	1.3 (68)	13.1 (704)	42.8 (2297)	42.8 (2301)	33.5 (1801)	66.5 (3569)
Osteoarthritis	0.2 (13)	2.1 (148)	32.6 (2316)	65.2 (4632)	42.5 (3022)	57.5 (4087)
Osteoporosis	0.3 (8)	1.5 (37)	26.0 (639)	72.1 (1770)	15.9 (391)	84.1 (2063)
Acquired movement disorders	12.8 (494)	16.6 (642)	34.7 (1346)	35.9 (1392)	41.0 (1589)	59.0 (2285)
Rheumatoid arthritis	2.7 (32)	9.2 (107)	43.2 (504)	44.9 (523)	40.0 (466)	60.0 (700)
Migraine and headache	8.6 (273)	27.4 (871)	42.0 (1333)	22.0 (698)	28.6 (908)	71.4 (2267)
Epilepsy	15.5 (136)	22.5 (197)	35.0 (305)	26.9 (236)	56.6 (496)	43.4 (380)
Mood disorders	6.1 (475)	26.7 (2083)	43.1 (3362)	24.2 (1887)	38.6 (3014)	61.4 (4793)
Other mental disorders	6.2 (228)	31.2 (1151)	42.3 (1562)	20.3 (750)	44.2 (1630)	55.8 (2061)
COPD and bronchitis	0.8 (21)	4.0 (105)	39.1 (1024)	56.1 (1469)	52.6 (1378)	47.4 (1241)
Asthma	23.4 (1441)	26.0 (1599)	32.6 (2004)	17.9 (1102)	49.0 (3012)	51.0 (3134)
Atopic dermatitis	44.0 (1819)	25.5 (1052)	19.7 (815)	10.8 (446)	46.3 (1913)	53.7 (2219)
Psoriasis	3.9 (85)	15.5 (336)	42.8 (928)	37.8 (820)	48.4 (1049)	51.6 (1120)
Thyroid diseases	1.9 (43)	10.9 (249)	41.9 (956)	45.3 (1032)	17.9 (407)	82.1 (1873)
Diabetes mellitus	1.0 (44)	3.6 (156)	33.7 (1479)	61.7 (2704)	56.0 (2456)	44.0 (1927)
Lipid metabolism disorder	0.4 (30)	3.9 (275)	41.3 (2920)	54.4 (3844)	50.2 (3548)	49.8 (3521)
Gout	0.8 (18)	3.7 (57)	35.3 (551)	60.7 (946)	79.5 (1239)	20.5 (320)
Urinary calculus	0.9 (17)	11.1 (236)	44.0 (934)	44.0 (934)	63.1 (1338)	36.9 (784)

Appendix 3a: Likelihood of cardiovascular diseases within other cardiovascular diseases

	Coronary artery diseases OR (95%CI)	Hypertension OR (95%CI)	Heart failure OR (95%CI)	Cerebrovascular diseases OR (95% CI)	Peripheral artery diseases OR (95%CI)
Diseases					
Peripheral artery disease	8.01 (7.12–9.01)	5.69 (5.17–6.26)	10.21 (8.35–12.47)	8.57 (7.57–9.70)	–
Cerebrovascular diseases	4.81 (4.27–5.43)	5.99 (5.50–6.53)	6.63 (5.38–8.17)	–	[a]
Heart failure	17.42 (14.76–20.54)	6.65 (5.67–7.80)	–	[a]	[a]
Hypertension	5.32 (4.92–5.75)	–	[a]	[a]	[a]
Coronary artery disease	–	[a]	[a]	[a]	[a]
Additional cardiovascular diseases	7.76 (7.17–8.40)	6.44 (6.10–6.81)	15.02 (12.38–18.23)	8.11 (7.42–8.86)	9.25 (8.36–10.24)

[a]Changing dependent and independent variables resulted in same odds ratios.
OR, odds ratio; 95%CI, 95% confidence interval.

Appendix 3b: Likelihood of chronic comorbid diseases within cardiovascular diseases

Chronic comorbid diseases	Coronary artery diseases OR (95%CI)	Hypertension OR (95%CI)	Heart failure OR (95%CI)	Cerebrovascular diseases OR (95%CI)	Peripheral artery diseases OR (95%CI)
Diseases					
All malignancies	3.05 (2.71–3.43)	3.21 (2.99–3.46)	4.23 (3.43–5.21)	3.05 (2.69–3.47)	2.94 (2.55–3.40)
Peptic ulcers	4.00 (3.38–4.74)	3.28 (2.91–3.69)	5.45 (4.09–7.27)	3.97 (3.31–4.77)	4.92 (4.08–5.94)
Other chronic gastrointestinal disease	3.11 (2.76–3.50)	3.49 (3.24–3.75)	2.95 (2.32–3.74)	2.97 (2.60–3.38)	2.75 (2.37–3.19)
Diseases of eye	4.61 (4.21–5.04)	5.40 (5.09–5.72)	8.30 (7.05–9.77)	4.75 (4.31–5.24)	5.08 (4.56–5.65)
Diseases of ear	3.84 (3.42–4.31)	3.51 (3.25–3.79)	4.66 (3.77–5.75)	3.82 (3.37–4.33)	2.97 (2.55–3.45)
Arrhythmias	2.55 (2.30–2.82)	4.96 (4.62–5.32)	23.51 (20.00–27.64)	5.29 (4.74–5.90)	4.58 (4.04–5.18)
Pulmonary circulatory disease	2.58 (2.13–3.13)	2.73 (2.42–3.08)	4.40 (3.24–5.98)	3.46 (2.87–4.18)	3.28 (2.65–4.06)
Diseases of veins	1.44 (1.27–1.63)	2.19 (2.06–2.34)	1.70 (1.34–2.15)	2.03 (1.80–2.30)	1.83 (1.59–2.11)
Osteoarthritis	3.62 (3.32–3.95)	4.87 (4.62–5.14)	4.65 (3.93–5.49)	3.54 (3.22–3.89)	3.82 (3.44–4.24)
Osteoporosis	1.97 (1.68–2.30)	3.21 (2.94–3.49)	4.08 (3.22–5.18)	3.35 (2.91–3.86)	3.03 (2.57–3.56)
Acquired movement disorders	1.38 (1.19–1.60)	1.79 (1.66–1.93)	1.32 (0.97–1.78)	1.78 (1.54–2.05)	1.87 (1.60–2.19)
Rheumatoid arthritis	2.27 (1.85–2.80)	2.71 (2.40–3.07)	4.03 (2.91–5.58)	2.92 (2.37–3.58)	2.41 (1.88–3.08)
Migraine and headache	0.94 (0.78–1.13)	1.53 (1.40–1.67)	0.57 (0.35–0.92)	1.64 (1.39–1.93)	1.01 (0.81–1.26)
Epilepsy	1.84 (1.42–2.38)	1.27 (1.07–1.50)	2.16 (1.33–3.52)	4.36 (3.55–5.34)	2.21 (1.65–2.97)
Mood disorders	1.50 (1.35–1.67)	1.57 (1.48–1.67)	1.67 (1.36–2.06)	1.78 (1.59–1.98)	1.56 (1.38–1.78)
Other mental disorders	1.43 (1.23–1.65)	1.22 (1.12–1.32)	1.59 (1.19–2.12)	1.57 (1.34–1.83)	1.55 (1.30–1.84)
COPD and bronchitis	4.60 (4.09–5.16)	3.25 (3.00–3.53)	7.94 (6.57–9.61)	3.98 (3.49–4.53)	6.47 (5.71–7.34)
Asthma	0.95 (0.83–1.09)	1.08 (1.00–1.15)	1.29 (1.01–1.66)	1.04 (0.90–1.21)	0.98 (0.83–1.16)
Atopic dermatitis	0.51 (0.41–0.63)	0.53 (0.48–0.59)	0.58 (0.38–0.89)	0.74 (0.60–0.90)	0.47 (0.36–0.62)
Psoriasis	2.29 (1.96–2.68)	2.19 (1.99–2.41)	2.68 (2.00–3.59)	2.14 (1.80–2.55)	2.47 (2.06–2.97)
Thyroid diseases	2.30 (1.98–2.68)	2.76 (2.52–3.02)	3.72 (2.89–4.79)	2.43 (2.07–2.86)	2.36 (1.97–2.84)
Diabetes mellitus	5.90 (5.39–6.47)	7.59 (7.12–8.09)	8.77 (7.43–10.36)	4.19 (3.77–4.66)	5.33 (4.77–5.96)
Lipid metabolism disorder	4.97 (4.57–5.40)	8.08 (7.66–8.52)	3.47 (2.91–4.14)	3.74 (3.41–4.12)	4.66 (4.21–5.16)
Gout	4.84 (4.20–5.57)	7.24 (6.53–8.02)	8.30 (6.64–10.37)	4.04 (3.44–4.74)	4.63 (3.91–5.49)
Urinary calculus	3.03 (2.62–3.49)	2.97 (2.71–3.25)	1.97 (1.41–2.76)	2.35 (1.98–2.79)	2.34 (1.93–2.82)

OR, odds ratio; 95%CI, 95% confidence interval.
